# Low Phospholipid-Associated Cholelithiasis: Contribution of Imaging in Two Cases

**DOI:** 10.7759/cureus.22383

**Published:** 2022-02-19

**Authors:** Fatima Zahra Belabbes, Amine Benfaida, Bouknani Nawal, Abdennaceur El Idrissi Lamghari, Fedoua Rouibaa

**Affiliations:** 1 Gastroenterology and Proctology, Faculty of Medicine, Mohammed VI University of Health Sciences (UM6SS) Cheikh Khalifa International University Hospital, Casablanca, MAR; 2 Radiology, Faculty of Medicine, Mohammed VI University of Health Sciences (UM6SS) Cheikh Khalifa International University Hospital, Casablanca, MAR

**Keywords:** imaging, lpac syndrome, cholangitis, lithiasis, intrahepatic

## Abstract

Low phospholipid-associated cholelithiasis (LPAC) is a genetic disease responsible for the development of intrahepatic lithiasis. It is retained in the presence of two of the following three criteria: age of onset of biliary symptoms less than 40 years; echogenic intrahepatic images or microlithiasis; and the recurrence of biliary clinical signs after cholecystectomy. The majority of clinical situations are simple and not serious, but some complicated forms may require more invasive endoscopic or surgical treatments. By presenting two case studies, we illustrated and summarized the different aspects of this entity.

## Introduction

Low phospholipid-associated cholelithiasis (LPAC) syndrome is a rare disease. It is a particular form of intrahepatic cholestasis that occurs in young adults. It is caused by an ATP-binding cassette subfamily B member 4 (ABCB4) mutation that codes for multidrug resistance protein 3 (MDR3), a biliary carrier. ABCB4 is an ATP-binding cassette membrane transporter that translocates phosphatidylcholine from the inner to the outer leaflet of the canalicular membrane of the hepatocyte by bile salts [1]. This genetic mutation has causally been linked to a defect of phospholipid canalicular secretion into bile, leading to impaired solubilization of biliary cholesterol that precipitates in the form of crystals in canaliculi and intrahepatic bile ducts [2].

Several complications have been described, the most common being extensive intrahepatic lithiasis, migration of the lithiasis, and acute cholangitis. A chronic course may lead to secondary sclerosing cholangitis or cirrhosis [3]. Diagnosis and treatment of weakly phospholipid-associated cholelithiasis (LPAC) syndrome are easy, but the majority of cases are underestimated because they go undiagnosed. Intrahepatic stones can be shown by ultrasound with color Doppler examination, computed tomography, and magnetic resonance imaging with magnetic resonance cholangiography, and diagnostic confirmation is made by genotypic ABCB4 [4].

We report these observations in two patients with LPAC syndrome, diagnosed after cholecystectomy and complicated by acute cholangitis after endoscopic and medical treatment.

## Case presentation

Case presentation 1

**Figure 1:**
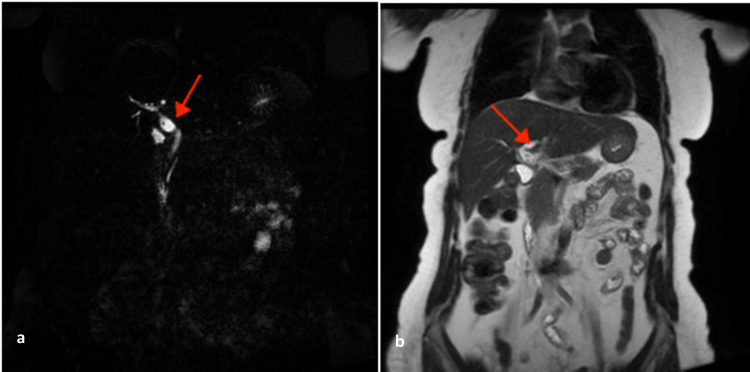
MRI with sequence heavily weighted in T2 (a) and coronal slice in T2 (b) showing segmental dilation of the main bile duct (red arrow) with endoluminal lithiasis. MRI: magnetic resonance imaging.

A 72-year-old woman who had a history of cholecystectomy presented with recurrent cholangitis accompanied by fever and chills. On clinical examination, she was febrile, and her conjunctivae and skin were icteric. She had an abnormal liver test. Cytolysis was observed in the presence of cholestasis, with aspartate aminotransferase (ASAT) of 250 IU/L, alanine aminotransferase (ALAT) of 370 IU/L, total and direct bilirubin levels, respectively, of 27 and 14.95 mg/dL, alkaline phosphatase (ALP) of 140 IU/L, and γ-glutamyltransferase (GGT) of 339 IU/L. An ultrasound examination showed the presence of lithiasis in the common bile duct, corresponding to residual lithiasis of the common bile duct. Cholangio-MRI confirmed the common bile duct dilatation with massive lithiasis into the extra and intrahepatic biliary ducts (Figure 1).

**Figure 2:**
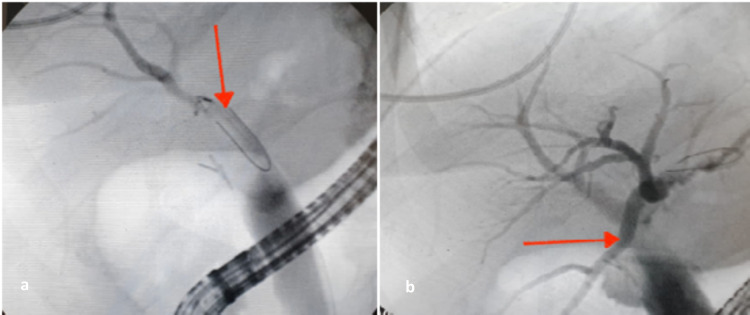
Endoscopic retrograde cholangiopancreatography showing: (a) stenosis of the main bile duct (red arrow) and (b) the disappearance of this stenosis (red arrow) after use of the stone extraction balloon.

Biliopancreatic endoscopy was performed two months later and showed dilation of the intra and extrahepatic bile duct with choledocious stones and thick biliary sludge (Figure 2).

Viral hepatitis serology, including hepatitis A, B, C, and E, Epstein-Barr virus, and cytomegalovirus, as well as human immunodeficiency virus (HIV), autoimmune status, in particular (antinuclear antibodies, anti-smooth muscle, antimitochondrial, anti-neutrophil, anti-gp210, anti-Sp100 cytoplasmic antibodies, and IgG4 level) and overload workup, including Wilson's disease and hemochromatosis, were normal.

**Figure 3:**
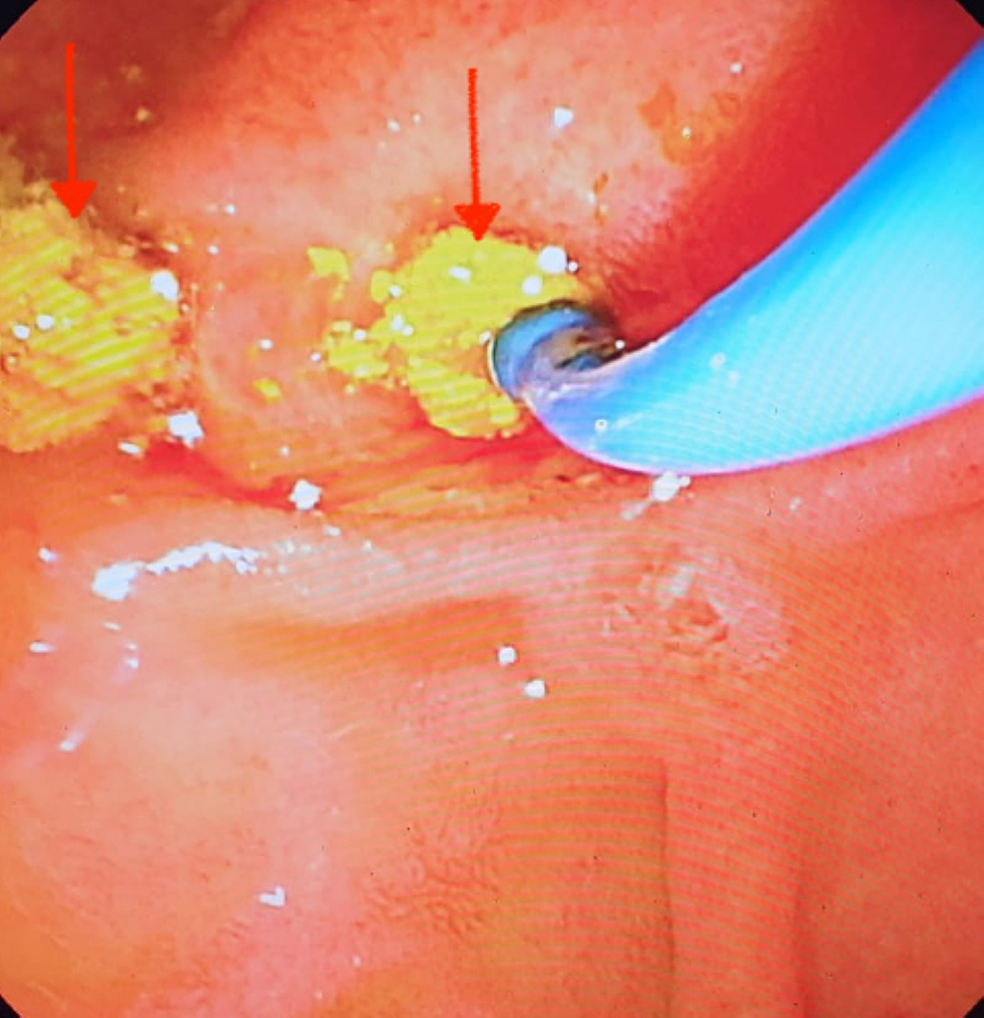
Retrograde cholangiopancreatography: endoscopic image after dilation showing the extraction of gallstones (red arrow).

A basic medical treatment with ursodeoxycholic acid was started with a regular biological control. The patient underwent endoscopic retrograde cholangiopancreatography with sphincterotomy and evacuation of calculus debris and biliary sludge (Figure 3).

All these therapeutic steps were conclusive in the short term, but each time a distant recurrence appears.

Case presentation 2

A 70-year-old man with cholecystectomy in the past, with a recurrence of painful seizures after five years. The diagnosis of LPAC syndrome was therefore confirmed, and the patient received UDCA medical treatment at a dose of 10 mg/kg with a slight decrease in abdominal pain. She was admitted for a recurrence of cholangitis.

**Figure 4:**
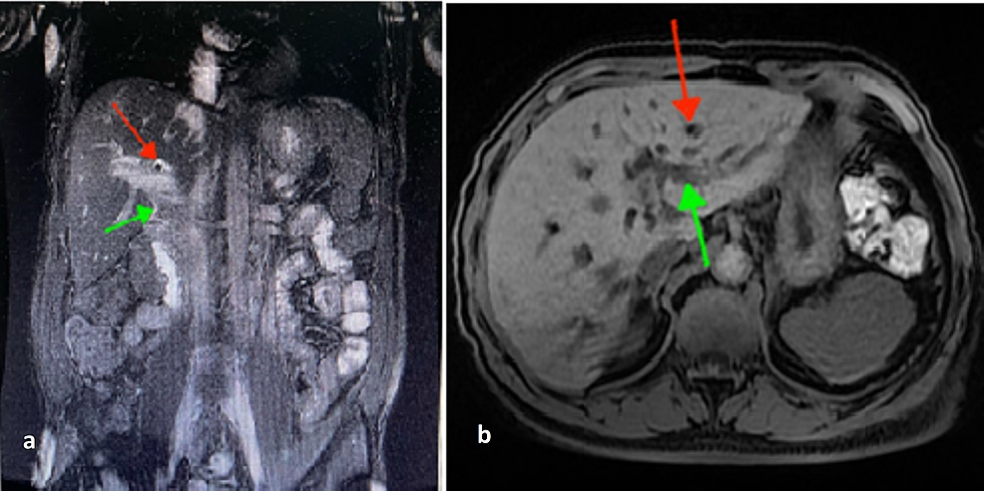
A 70-years-old patient with cholangitis: MRI with coronal T2 (a) and axial T1 (b) slice showing lithiasis in the absence of signal from the intrahepatic bile ducts (red arrow) with segmental dilations (green arrow). MRI: magnetic resonance imaging.

**Figure 5:**
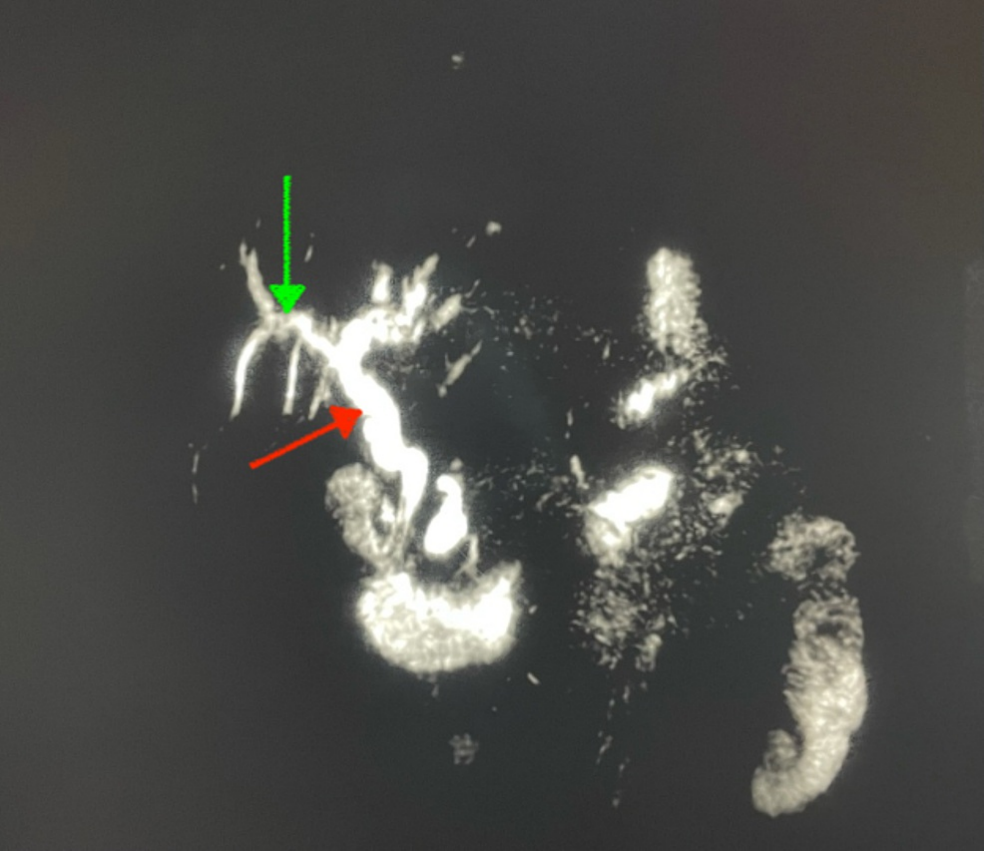
A 70-years-old patient with cholangitis: 3D MRI sequences and showing segmental dilation (red arrow) of the intra and extrahepatic bile ducts (green arrow). MRI: magnetic resonance imaging.

An ultrasound examination showed the presence of lithiasis in the common bile duct, evoking residual lithiasis of the common bile duct. The cholangio magnetic resonance imaging (MRI) confirmed the common bile duct dilatation (Figures 4-5).

**Figure 6:**
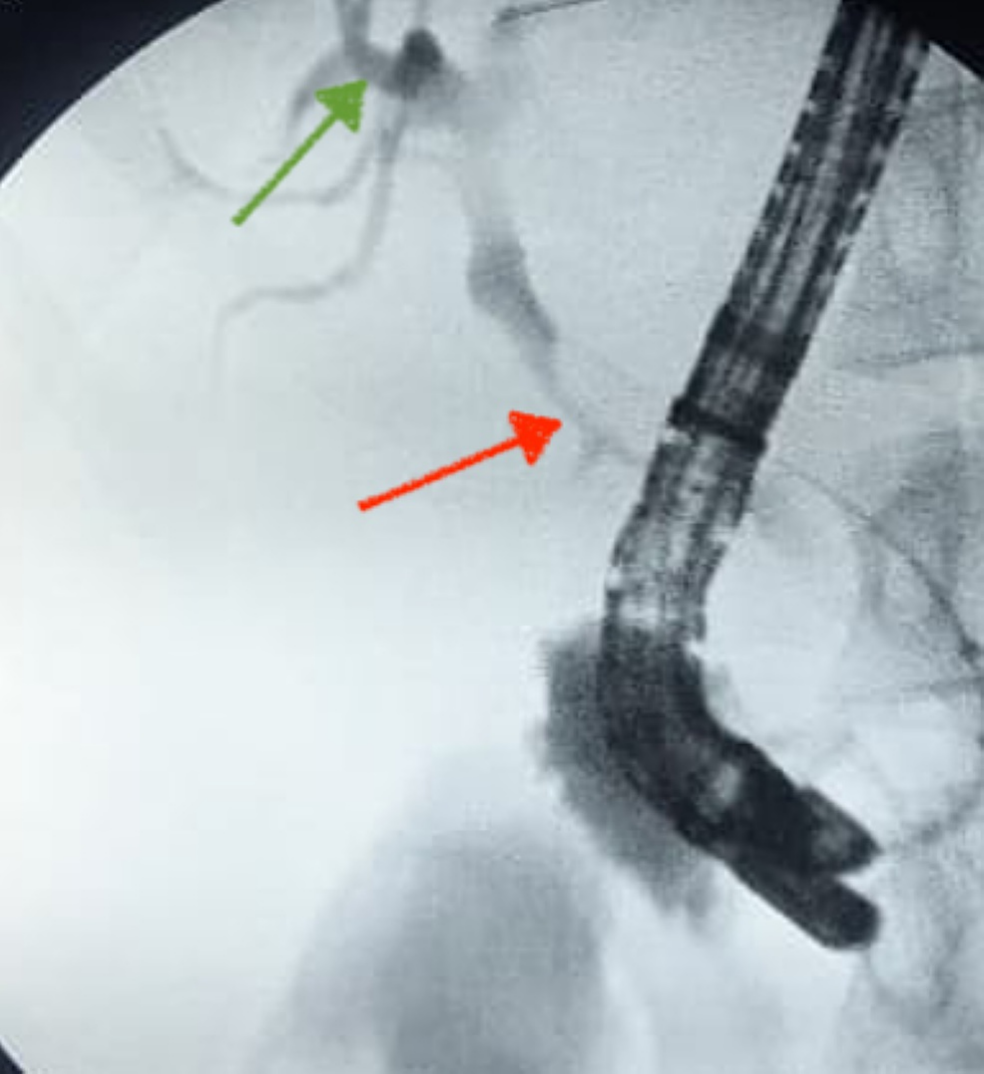
Endoscopic retrograde cholangiopancreatography: X-ray image after opacification showing stenosis of the main bile duct (red arrow) with dilation of the intrahepatic bile ducts (green arrow).

The opacification during the endoscopic retrograde cholangiopancreatography showed moderately dilated extrahepatic bile ducts, upstream of a stenosis of the lower bile duct (Figure 6).

The chosen therapeutic option was the installation of two plastic prostheses with a good emptying of the bile ducts as a result, and, of course, the maintenance of ursodeoxycholic acid as a basic medical treatment. The patient improved clinically, with the resolution of pain, fever, and jaundice.

## Discussion

LPAC is a genetic disease responsible for the formation of intrahepatic stones. It is characterized by the association of ATP-binding cassette subfamily B member4 (ABCB4) and low bile phospholipids with symptomatic and recurrent cholelithiasis. The exact prevalence of LPAC remains unknown [5]. The average age of onset of symptoms is 26 to 32 years. The male to female ratio is estimated at approximately 1:3 [6]. The onset of symptoms typical of LPAC syndrome has been reported during pregnancy, either in the final stages or after delivery [7]. It is a very particular form of the lithiasis gall bladder which was first described in 2001 by the Saint-Antoine Hospital [7].

The phospholipid transporter (MDR3 protein, encoded by the gene ABCB4) allows the excretion of phospholipids in the bile at the canalicular pole of hepatocytes. Mutations in the ABCB4 gene have been logically identified as responsible for LPAC syndrome, which is involved in biliary phosphatidylcholine excretion. However, these mutations are observed in only approximately 60% of cases (in a subset of patients) [2].

LPAC syndrome is an exclusive clinical entity. The most common form of presentation of LPAC syndrome is an evocative picture of migration lithiasis (biliary pain associated with a fleeting increase in transaminase). The presence of intrahepatic gallstones and elevated serum gamma-glutamyl transferase activity are frequently present in LPAC syndrome [8].

Although some patients will only report rare episodes of pain in the gallbladder, most untreated patients will have severe and recurrent symptoms. The diagnosis is made in less than half of the cases. Radiology plays a major role in the diagnosis of this condition. It is made by an expert in ultrasound (a sensitized radiologist). Ultrasound signs are associated with the appearance of comet tails with cholesterol deposits and intrahepatic bile duct microlithiasis. Hepatic bile content is obtained by duodenoscopy and aspiration using a catheter introduced into the distal bile duct or by T-tube after surgery. Most often, these pathognomonic radiological signs of LPAC syndrome are not detected by entero-computed tomography (CT) or magnetic resonance cholangiopancreatography (MRCP).

However, in supplement to this ultrasound expert, a Bili-MRI is necessary to eliminate differential diagnoses, including primary sclerosing cholangitis and Caroli's disease. The ABCB4 genotype must be requested to confirm the diagnosis of LPAC syndrome in young adults with symptomatic cholelithiasis; it also allows family screening [9]. The diagnosis of LPAC syndrome should be considered when at least two of the following criteria are met: age less than 40 years at the onset of clinical signs; recurrence of biliary symptoms after cholecystectomy; intrahepatic hyperechoic images; or sludge or microlithiasis along the bile ducts [8]. The genetic diagnosis is based on the demonstration of a significant mutation in the ABCB4 gene. However, ABCB4 mutations were only found in one-third to one-half of the LPAC [10].

Treatment of LPAC syndrome is based on prolonged treatment with AUDC at a dosage of 10 mg/kg/day. The effectiveness of medical treatment with AUDC is such that cholecystectomy can be avoided in the majority of cases. Indeed, complicated forms may require interventional radiological, endoscopic, or even surgical treatment. Evacuation by biliary or partial hepatectomy can be proposed in the case of dilation of the intrahepatic bile ducts filled with gallstones with the persistence of symptoms. [4]. The resection of the segment or lobe affected may be justified if the patient presents with recurrent cholangitis despite treatment with AUDC, but it does not prevent the recurrence of symptoms. Family screening concerns adult relatives of the first degree [3]. Family screening by an ultrasound expert (and/or genotypic if an ABCB4 mutation has been demonstrated in a proband) may be offered to parents of first degree children over 18 years old.

## Conclusions

Through the presentation of our observations in the light of contemporary literature, we have tried to take stock of the clinical and radiological manifestations, allowing us to retain an LPAC syndrome, to facilitate early diagnosis, and to improve the management as well as the evolution of this pathology. The ABCB4 genotype should be increasingly requested by practitioners to make a positive diagnosis of LPAC syndrome in young people with cholelithiasis symptoms and for family screening. Several studies have shown that most cases of LPAC syndrome are undiagnosed. This underdiagnosis is partly explained by the lack of knowledge of a disease by the various gastroenterologists and radiologists.
